# Enhancing Type 2 Diabetes Treatment Decisions With Interpretable Machine Learning Models for Predicting Hemoglobin A1c Changes: Machine Learning Model Development

**DOI:** 10.2196/56700

**Published:** 2024-07-18

**Authors:** Hisashi Kurasawa, Kayo Waki, Tomohisa Seki, Akihiro Chiba, Akinori Fujino, Katsuyoshi Hayashi, Eri Nakahara, Tsuneyuki Haga, Takashi Noguchi, Kazuhiko Ohe

**Affiliations:** 1 Nippon Telegraph and Telephone Corporation Tokyo Japan; 2 The University of Tokyo Hospital Tokyo Japan; 3 NTT DOCOMO, Inc Tokyo Japan; 4 NTT-AT IPS Corporation Kanagawa Japan; 5 National Center for Child Health and Development Tokyo Japan

**Keywords:** AI, artificial intelligence, attention weight, type 2 diabetes, blood glucose control, machine learning, transformer

## Abstract

**Background:**

Type 2 diabetes (T2D) is a significant global health challenge. Physicians need to assess whether future glycemic control will be poor on the current trajectory of usual care and usual-care treatment intensifications so that they can consider taking extra treatment measures to prevent poor outcomes. Predicting poor glycemic control from trends in hemoglobin A_1c_ (HbA_1c_) levels is difficult due to the influence of seasonal fluctuations and other factors.

**Objective:**

We sought to develop a model that accurately predicts poor glycemic control among patients with T2D receiving usual care.

**Methods:**

Our machine learning model predicts poor glycemic control (HbA_1c_≥8%) using the transformer architecture, incorporating an attention mechanism to process irregularly spaced HbA_1c_ time series and quantify temporal relationships of past HbA_1c_ levels at each time point. We assessed the model using HbA_1c_ levels from 7787 patients with T2D seeing specialist physicians at the University of Tokyo Hospital. The training data include instances of poor glycemic control occurring during usual care with usual-care treatment intensifications. We compared prediction accuracy, assessed with the area under the receiver operating characteristic curve, the area under the precision-recall curve, and the accuracy rate, to that of LightGBM.

**Results:**

The area under the receiver operating characteristic curve, the area under the precision-recall curve, and the accuracy rate (95% confidence limits) of the proposed model were 0.925 (95% CI 0.923-0.928), 0.864 (95% CI 0.852-0.875), and 0.864 (95% CI 0.86-0.869), respectively. The proposed model achieved high prediction accuracy comparable to or surpassing LightGBM’s performance. The model prioritized the most recent HbA_1c_ levels for predictions. Older HbA_1c_ levels in patients with poor glycemic control were slightly more influential in predictions compared to patients with good glycemic control.

**Conclusions:**

The proposed model accurately predicts poor glycemic control for patients with T2D receiving usual care, including patients receiving usual-care treatment intensifications, allowing physicians to identify cases warranting extraordinary treatment intensifications. If used by a nonspecialist, the model’s indication of likely future poor glycemic control may warrant a referral to a specialist. Future efforts could incorporate diverse and large-scale clinical data for improved accuracy.

## Introduction

Type 2 diabetes (T2D) affects an estimated 529 million people globally [1]. Hemoglobin A_1c_ (HbA_1c_) serves as an indicator of poor glycemic control, reflecting the average blood glucose levels over 1 to 2 months. An increase in HbA_1c_ of 1 percentage point worsens cardiovascular disease risk by 1.2 times and mortality risk by 1.14 times [2]. According to the American Diabetes Association *Standards of Care in Diabetes* [3], target HbA_1c_ levels are set at 7% for many adults who are nonpregnant and 8% for patients with limited life expectancy or where the harms of treatment are greater than the benefits.

Physicians need to identify early signs of impending poor glycemic control in patients with T2D and act early to intensify treatment, via a combination of pharmacological and lifestyle interventions, to avoid poor outcomes. There are costs to intensified treatment, including side effects, so it is prudent to delay intensification until it is warranted by disease progression. Factors associated with poor glycemic control include age, duration of T2D treatment, treatment, race or ethnicity, and family history [4-7]. External factors such as seasonal variations affecting HbA_1c_ levels [8] complicate accurate glycemic control prediction.

People with T2D receive care from primary care physicians, not T2D specialists, in many areas including the United States, Europe [9], and Japan [10]. For example, two-thirds of people with T2D in Japan receive care from primary care physicians [10]. These nonspecialists may struggle to predict a patient’s glycemic control. In Japan, approximately 60% of surveyed patients with T2D treated by nonspecialists experienced poor glycemic control (HbA_1c_≥8%), with around 30% seeing worsened levels the following year, according to a survey on T2D treatment practices by primary care physicians [10].

Physicians regularly adjust a T2D patient’s treatment, intensifying treatment when the clinical indications lead them to predict poor glycemic control. Despite this usual care, including treatment intensification, some patients still experience poor glycemic control. From 2015 to 2018, a total of 49.5% of US community-dwelling adults with diabetes had HbA_1c_≥7% and 24.6% had HbA_1c_≥8% [11]. A tool predicting poor glycemic control while under usual care, including usual-care treatment intensifications, could enhance treatment outcomes. It could alert physicians early enough to enable intensified modification of treatment, improving treatment outcomes for patients and increasing referrals to specialists when warranted by disease progression.

Machine learning (ML) has demonstrated success in predicting patient symptoms, including forecasting the onset of T2D [12] and predicting complications [13], and it is a promising approach to predicting poor glycemic control, although to our knowledge it has not previously been applied to this task. Glycemic control data are in general irregularly spaced, reflecting the variability in patient care appointment dates, with updates to outpatient electronic health records (EHRs) occurring before and after clinical visits. Irregularly spaced data require preprocessing techniques such as interpolation, denoising autoencoders, and self-supervised learning [14-17]. Processing data with irregular intervals may hurt predictive performance [18], requiring careful consideration in developing artificial intelligence models.

Although ML models may provide good prediction performance, they often operate as “black boxes,” with opaque reasoning and associated poor interpretability that makes it difficult for both physicians and patients to understand the logical process guiding decision-making [19]. To allow the interpretation of ML models, so that they are more acceptable to physicians [20,21] and patients [22], explainable artificial intelligence (XAI) has been studied [23]. It attempts to clarify temporal relationships of symptoms at each time point toward temporal interpretability based on patient trajectories [24,25], and this has been actively researched in the computer science field [26].

Since its introduction in 2017, the transformer model has excelled in various time-series predictive tasks, solidifying its position as a core technology across multiple fields [27-32]. The transformer model incorporates an attention mechanism simplifying the extraction of temporal relationships and setting it apart from other models [33-35]. The attention mechanism allows a model to selectively focus on different data points in the input sequence, assigning varying degrees of importance to each data point. Applied to the problem of predicting poor glycemic control, the attention mechanism can process irregularly spaced HbA_1c_ time series and quantify temporal relationships of past HbA_1c_ levels at each time point, following a model-specific approach in XAI [36].

This study aims to develop an ML tool that accurately and interpretably predicts poor glycemic control (HbA_1c_≥8%) using irregularly spaced HbA_1c_ levels over the past year, in support of preventing T2D complications by enabling timely intensification of treatment. Although the treatment guidelines generally target an HbA_1c_ level of 7% or lower [3], higher levels are common in diabetes patients. In our clinical experience, levels of 8% and higher are a cause of great concern and trigger more intensive intervention. Accordingly, we have set 8% HbA_1c_ as the threshold for defining poor glycemic control.

Given the absence of prior studies in this specific area, we set target accuracy to be the receiver operating characteristic (ROC) area under the curve (AUC)>0.9 and precision-recall (PR)–AUC>0.8 based on our clinical endocrinology experience with diabetes treatment. These values are commonly used as a benchmark for good prediction accuracy in the ML field [37] and are consistent with the ROC-AUCs of past diabetes-related ML tasks ranging from 0.819 to 0.934 [38-42].

Drawing on our team’s prior work in self-management support for T2D treatment [43] and predicting treatment discontinuations [44,45], we designed this task with the hope of overcoming barriers to implementing ML in clinical practice, believing it could significantly advance T2D diagnosis and treatment.

We hypothesize that an ML model can predict poor glycemic control in patients with T2D under usual care. Our specific research question is whether a transformer-based model, incorporating temporal relationships of HbA_1c_ levels, can accurately and interpretably predict instances of poor glycemic control (HbA_1c_≥8%). Our approach is novel in how it overcomes challenges posed by irregularly spaced HbA_1c_ time series.

## Methods

### Data Sets and Preprocessing

All data were collected from EHRs at the University of Tokyo Hospital, which included 7787 patients who visited the hospital and had diagnostic codes indicative of T2D. The data were recorded in the EHRs between January 1, 2006, and December 31, 2015. The data, including treatment decisions and outcomes, were reflective of care by T2D specialists. Only HbA_1c_ levels were used in the ML model.

### ML Models

Given the irregularly spaced data, we organized the data into Monday-to-Sunday weeks and quantized the data to a single value per week, using the average in the case of multiple measurements and treating weeks with no values as having missing values [46]. This approach allowed the ML model to treat irregularly spaced data spanning N years as regularly spaced data consisting of N×365/7 (rounded up to the nearest integer) values, that is, we treat all data as weekly data. We did not perform preprocessing, including interpolation, on missing values in the regularly spaced data. No normalization, outlier removal, or dimensionality reduction were performed on the HbA_1c_ levels. Typical ML models such as LightGBM address missing values via interpolation or replacement before learning. In contrast, we adopted an approach that ignores and skips missing values.

**Figure 1:**
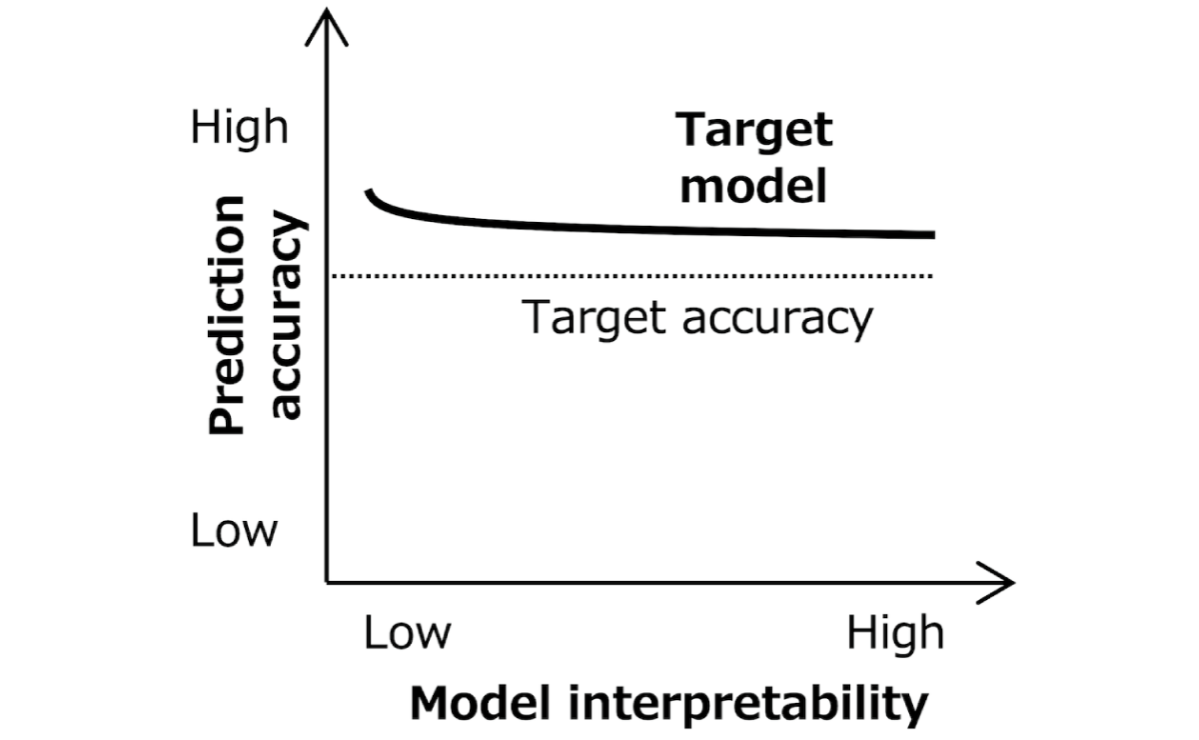
Conceptual trade-off between prediction accuracy and interpretability, for a given level of computational complexity.

We designed a transformer model (Table 1) that takes as input an irregularly spaced time series of HbA_1c_ levels spanning over the past year or more and outputs a binary assessment of poor glycemic control (HbA_1c_≥8%) within the subsequent year. The model incorporates 2 types of attention layers: self-attention, designed to extract temporal relationships from past irregularly spaced HbA_1c_ levels, and cross-attention, used to predict poor glycemic control based on these temporal relationships. The self-attention weights are optimized through self-supervised learning. This involves the task of predicting the next HbA_1c_ level using a time series of weekly spaced past HbA_1c_ levels with missing values, where the past levels are used as both input and output. We use an attention mask mechanism that completely ignores missing values by setting their self-attention weight to 0, allowing us to learn using values with irregular spacing due to missing values as is. This is similar to the process of padding in language models. The cross-attention weights are optimized through supervised learning. This involves the task of predicting the class representing the likelihood of future poor glycemic control using the latent variables transformed by self-attention from the past HbA_1c_ time series. We used causal masking in both learning tasks to prevent the model from referencing future data, ensuring that the model makes predictions considering the causal relationship between past symptoms and future symptoms. Conceptually, given that we constrained the model to improve interpretability, we expect a slightly lower prediction accuracy than that of an unconstrained model (Figure 1), and 1 goal is to minimize this interpretability penalty.

**Table 1:** Model details (transformer architecture).

Configure	Value
Encoder layers including self-attention blocks, n	4
Decoder layers including cross-attention blocks, n	4
Heads in the attention, n	4
Transformer hidden size, n	128
Transformer feedforward neural network hidden size, n	512
Optimizer method	Adam
Loss function	Focal Loss
Learning rate	1×10^–4^
Batch size, n	512
Iterations, n	20,000 (no early stopping)
Library	Python (version 3.11) and PyTorch (version 2.2.0)

This model makes a yes or no decision on future poor glycemic control, with a threshold as a free variable allowing a tradeoff among true or false negatives or positives. We set the threshold to maximize the *F*_1_-score (the harmonic mean of the ROC and PR values) using training data, resulting in a tool that made a binary prediction as to whether or not, in the next year, glycemic control will be poor. The training data include treatment intensification by specialists making their own assessments of likely future glycemic control. As such, the predicted poor glycemic control occurs despite any usual-care intensification of treatment prescribed by the attending specialist physicians. In other words, a prediction of poor glycemic control indicates a case likely warrants special attention and intervention, as usual-care intensification of treatment is predicted to be insufficient.

### Temporal Data Usage

Our analysis sought to determine the length of the HbA_1c_ time series needed to achieve the target accuracy. Training and testing were separated by period using the well-established time series prediction accuracy evaluation method [47]. We used as a reference the date on which a patient took an HbA_1c_ test in 2013. We used the HbA_1c_ time series for the N years before the reference date as training input and the occurrence or absence of poor glycemic control (HbA_1c_≥8%) within 1 year from the reference date as the training output. Then, we tested the resulting model using the same procedure, but for the following year, 2014, selecting an appropriate choice for N, the length of training data. We evaluated the predictive performance of the resulting model using 7 years of test data, sliding the reference dates from 2007 to 2013, using the rolling-origin procedure [47].

The training input or output period and the testing output period do not overlap, and therefore there was no leakage into predictive evaluation. Data for a given patient will in general have some time samples in the training data and some in the test data, but since patient identification is not an input to the model, the model does not identify specific patients.

### Statistical Methods

We analyzed the characteristics of patients in the data set using means, SDs, and frequency counts. We performed all statistical analyses using custom Python code. We used the *Python* (version 3.11) and *PyTorch* (version 2.2) libraries for developing the transformer model, the *Numpy* (version 1.26) and *Pandas* (version 2.2) libraries for managing data sets, and the *scikit-learn* (version 1.4) library for evaluating predictive accuracy.

We compared our model with an established ML method recognized for high accuracy. There were no studies directly addressing our task, but validations on similar T2D prediction tasks favored LightGBM [48,49], making it our chosen reference for comparisons. While LightGBM is acknowledged for its superior predictive performance, it is not inherently interpretable. The model’s complexity and intricate decision tree paths make it difficult to provide a straightforward interpretation of its predictions. Our reference LightGBM model takes as input equally spaced HbA_1c_ data and outputs a binary assessment of poor glycemic control (HbA_1c_≥8%).

We compared the transformer model and LightGBM using the evaluation metrics of ROC-AUC, PR-AUC, accuracy rate, and *F*_1_-score, with 95% CI using the bootstrap method.

### Ethical Considerations

This study was approved by the Institutional Review Board of the University of Tokyo School of Medicine (10705-(3)) and was conducted per the Declaration of Helsinki. This was a retrospective, noninterventional database study without patient involvement. Confidentiality was safeguarded by the University of Tokyo Hospital. According to the Guidelines for Epidemiological Studies of the Ministry of Health, Labour and Welfare of Japan, written informed consent was not required. Information about this study was available to patients on a website, and patients have the right to cease registration of their data at any time [50].

## Results

### Patient Data

We analyzed 7787 patients (Table 2). Although specialist physicians were providing usual care and prescribing treatment intensifications based on their clinical judgment, 57.83% (n=4504) of patients had an HbA_1c_ over 8% at least once. The number of HbA_1c_ tests per year was 7.7 (SD 2.8). In other words, the missingness level of weekly spaced past HbA_1c_ levels for a year was 1 – 7.7 / ROUNDUP(365/7) = 85.5%. The age group with the highest number of individuals is the aged 70-80 years category, comprising 2347 people, accounting for 30.14% of the patients. In addition to diabetes, more than 45% of patients had diseases such as essential (primary) hypertension, hypertensive heart disease, pure hypercholesterolemia, and astigmatism. Each patient had multiple records, leading to 323,825 records used in our analysis.

**Table 2:** Characteristics of patients.

Characteristics				Records (n=323,825)		Patients (n=7787)
**Feature used in the model**						
	**HbA_1c_^a^**					
		Mean (SD)	7.1 (1.1)		—^b^	
		<6%, n (%)	42,495 (13.12)		4103 (52.69)	
		6%-7%, n (%)	137,968 (42.61)		6666 (85.6)	
		7%-8%, n (%)	89,875 (27.75)		5770 (74.1)	
		≥8%, n (%)	53,487 (16.52)		4504 (57.84)	
	Tests per year, mean (SD)		7.7 (2.8)		—	
**Features not used in the model**						
	**Gender**					
		Male, n (%)	193,976 (59.9)		4726 (60.7)	
		Female, n (%)	129,849 (40.1)		3061 (39.3)	
	**Age (years)**					
		Mean (SD)	—		67.5 (13.6)	
		10-20, n (%)	—		1 (0.01)	
		20-30, n (%)	—		58 (0.74)	
		30-40, n (%)	—		255 (3.27)	
		40-50, n (%)	—		585 (7.51)	
		50-60, n (%)	—		1006 (12.92)	
		60-70, n (%)	—		2058 (26.43)	
		70-80, n (%)	—		2347 (30.14)	
		80-90, n (%)	—		1322 (16.98)	
		90-100, n (%)	—		149 (1.91)	
		100-110, n (%)	—		6 (0.08)	
	**Top 10 most common diseases**					
		E14: unspecified diabetes mellitus, n (%)	—		5495 (70.57)	
		I10: essential (primary) hypertension, n (%)	—		5023 (64.5)	
		E11: hypertensive heart disease, n (%)	—		3715 (47.71)	
		E780: pure hypercholesterolemia, n (%)	—		3661 (47.01)	
		H522: astigmatism, n (%)	—		3636 (46.69)	
		E785: hyperlipidemia, unspecified, n (%)	—		3490 (44.82)	
		K590: constipation, n (%)	—		3353 (43.06)	
		K210: gastro-esophageal reflux disease with esophagitis, n (%)	—		2937 (37.72)	
		K295: chronic gastritis, unspecified, n (%)	—		2756 (35.39)	
	**Top 10 most common medicines**					
		Metformin hydrochloride, n (%)	—		2541 (32.63)	
		Sitagliptin phosphate hydrate, n (%)	—		2177 (27.96)	
		Glimepiride, n (%)	—		2036 (26.15)	
		Pioglitazone hydrochloride, n (%)	—		1641 (21.07)	
		Insulin glargine (genetical recombination), n (%)	—		1597 (20.51)	
		Rosuvastatin calcium, n (%)	—		1458 (18.72)	
		Voglibose, n (%)	—		1430 (18.36)	
		Atorvastatin calcium hydrate, n (%)	—		1323 (16.99)	
		Insulin aspart (genetical recombination), n (%)	—		1277 (16.4)	
		Vildagliptin, n (%)	—		1187 (15.24)	

^a^HbA_1c_: hemoglobin A_1c_.

^b^Not applicable.

### Prediction Performance for HbA1c Time Series Lengths

**Figure 2:**
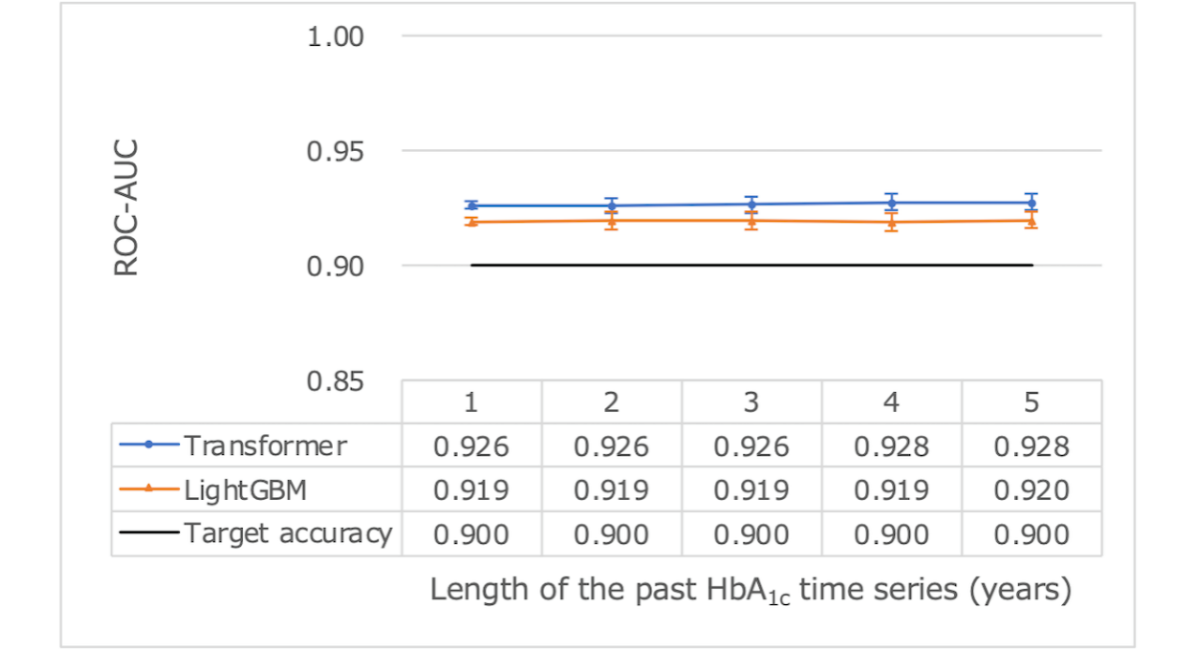
Predictive performance using ROC-AUC as a measure for various HbA_1c_ time series lengths using test data reference dates in 2014. HbA_1c_: hemoglobin A_1c_; ROC-AUC: area under the receiver operating characteristic curve.

**Figure 3:**
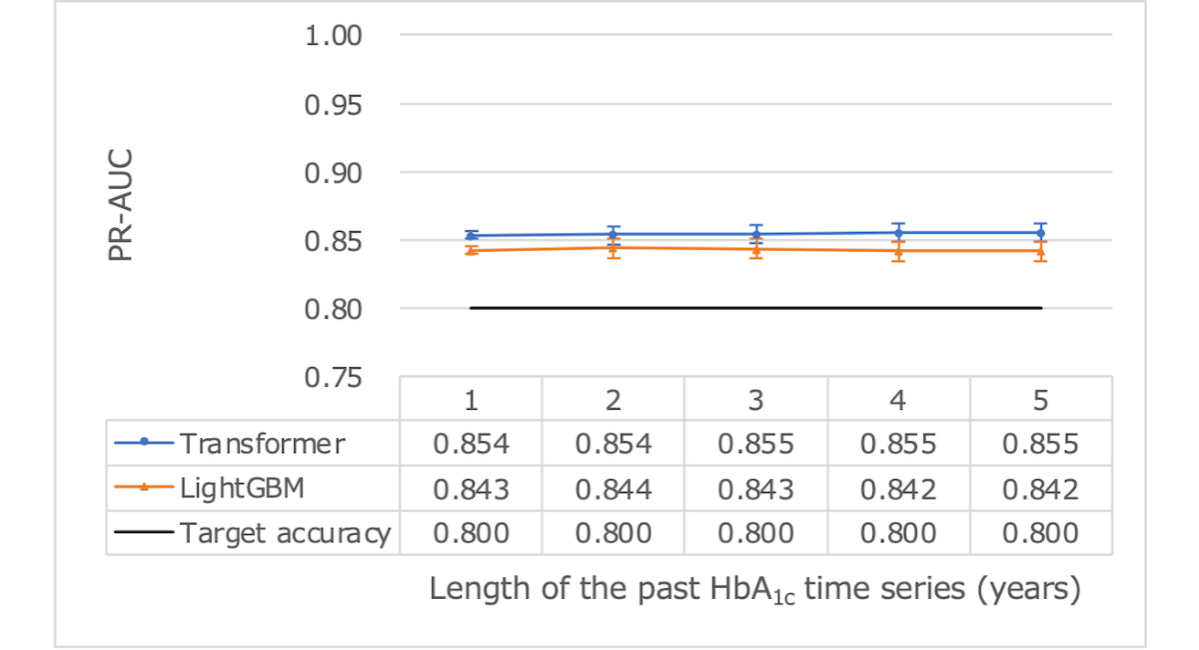
Predictive performance using PR-AUC as a measure for various HbA1c time series lengths using test data reference dates in 2014. HbA_1c_: hemoglobin A_1c_; PR-AUC: area under the precision-recall curve.

We assessed using different lengths of past HbA_1c_ time series (Table 3) as both training and test inputs to the model to determine the most effective period for predicting poor glycemic control. Extending the input period beyond 1 year did not yield a statistically significant difference within a 95% CI (Figures 2 and 3). This study’s objectives of achieving ROC-AUC>0.9 and PR-AUC>0.8 were attainable with just 1 year of past HbA_1c_ time series. Comparing prediction accuracy with LightGBM revealed no significant differences within the 95% CI, indicating nearly equivalent performance between the transformer and LightGBM. As a result, we settled on a final model that is based on using 1 year of prior data for training.

**Table 3:** Test data set size for the evaluation of various hemoglobin A_1c_ (HbA_1c_) time series lengths.

Length of past HbA_1c_ time series	Records (R), n	Records with poor glycemic control (T), n	T/R, %	Patients, n	Records per patient, mean (SD)	Weekly spaced data with values in input data, mean (SD)
1	25,564	6818	26.7	4661	5.5 (2.7)	7.3 (2.7)
2	25,594	6827	26.7	4672	5.5 (2.7)	13.2 (5.6)
3	25,611	6831	26.7	4676	5.5 (2.7)	18.8 (8.6)
4	25,618	6831	26.7	4678	5.5 (2.7)	24.1 (11.8)
5	25,621	6832	26.7	4678	5.5 (2.7)	28.9 (15.1)

### Prediction Performance Over the Full Data Set

**Figure 4:**
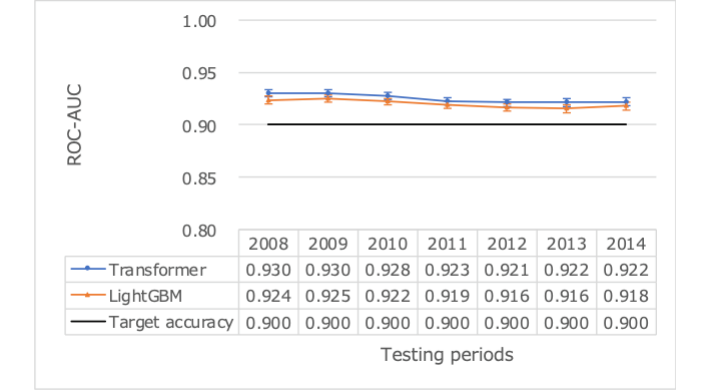
Predictive performance over time using ROC-AUC as a measure using test data reference dates ranging from 2008 to 2014. ROC-AUC: area under the receiver operating characteristic curve.

**Figure 5:**
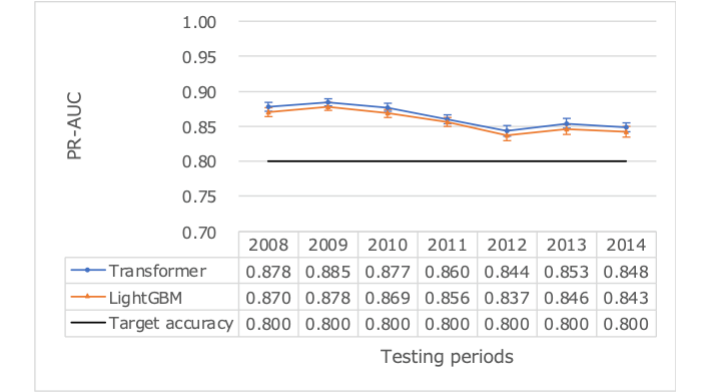
Predictive performance over time using PR-AUC as a measure using test data reference dates ranging from 2008 to 2014. PR-AUC: area under the precision-recall curve.

**Figure 6:**
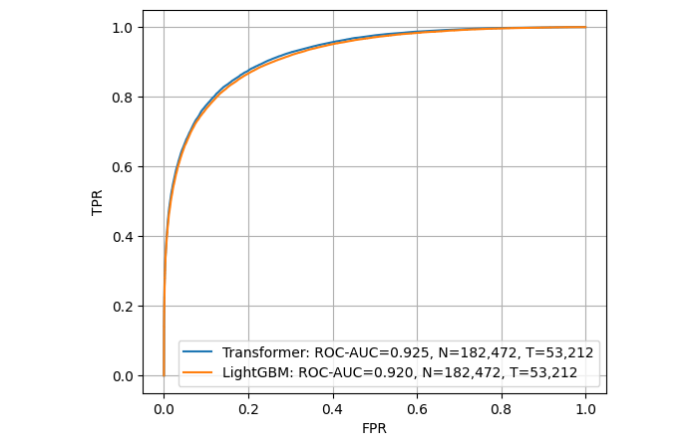
Predictive performance over time using ROC curve as a measure using test data reference dates ranging from 2008 to 2014. AUC: area under the curve; FPR: false positive rate; HbA_1c_: hemoglobin A_1c_; ROC: receiver operating characteristic; TPR: true positive rate.

**Figure 7:**
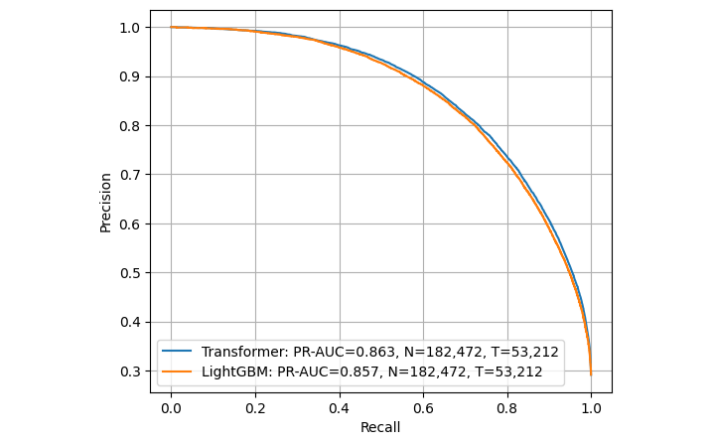
Predictive performance over time using PR curve as a measure using test data reference dates ranging from 2008 to 2014. AUC: area under the curve; HbA_1c_: hemoglobin A_1c_; PR: precision-recall.

We assessed whether the resulting model, using 1 year of prior data for training, could consistently achieve the target accuracy over the available 7 years of test data (Table 4). Despite some fluctuation in prediction accuracy, the target was achieved over the entire 7-year period (Figures 4 and 5). The ROC-AUC (95% confidence limits) for transformer was 0.925 (95% CI 0.923-0.928; Figure 6), compared to LightGBM’s 0.920 (95% CI 0.918-0.923), and the PR-AUC (95% confidence limits) for transformer was 0.864 (95% CI 0.852-0.875; Figure 7), compared to LightGBM’s 0.857 (95% CI 0.846-0.868). The average accuracy rate (95% confidence limits) for the transformer was 0.864 (95% CI 0.860-0.869), comparable to LightGBM’s 0.861 (95% CI 0.857-0.865).

**Table 4:** Test data set size for the evaluation of various hemoglobin A_1c_ (HbA_1c_) time series lengths.

Year of the test data	Records (R), n	Records with poor glycemic control (T), n	T/R, %	Patients, n	Records per patient, mean (SD)	Weekly spaced data with values in input data, mean (SD)
2007	22,520	7176	31.9	3221	7 (3.1)	8 (2.9)
2008	24,775	7517	30.3	3626	6.8 (3.1)	8.1 (2.9)
2009	26,144	8444	32.3	2973	6.6 (3)	8 (2.9)
2010	27,124	8521	31.4	4260	6.4 (3)	7.8 (2.9)
2011	26,661	7687	28.8	4377	6.1 (3)	7.7 (2.8)
2012	26,259	6944	26.4	4412	6 (2.9)	7.5 (2.7)
2013	25,945	7281	28.1	4533	5.7 (2.8)	7.4 (2.7)
2014	25,564	6818	26.7	4661	5.5 (2.7)	7.3 (2.7)

### Interpretability

**Figure 8:**
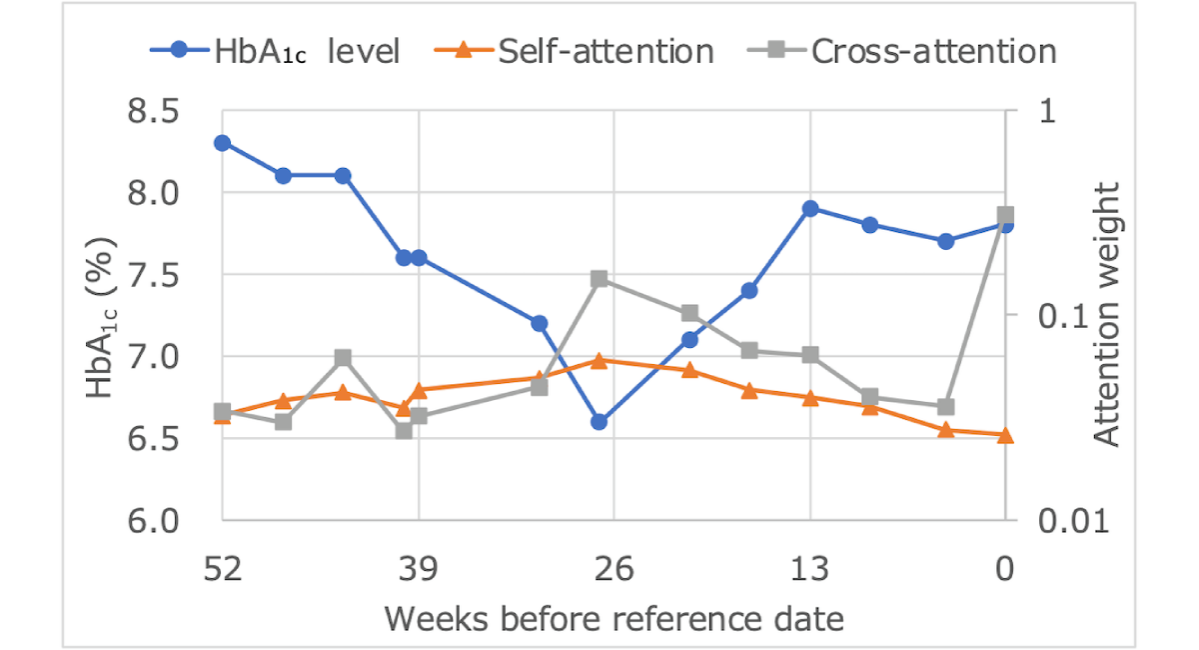
Example of HbA_1c_ levels, self-attention weights, and cross-attention weights. HbA_1c_: hemoglobin A_1c_.

The proposed model extracts temporal relationships from past irregularly spaced HbA_1c_ levels using self-attention and determines the contribution of each HbA_1c_ level to the prediction of glycemic control using cross-attention. An example of the extracted results is shown in Figure 8.

**Figure 9:**
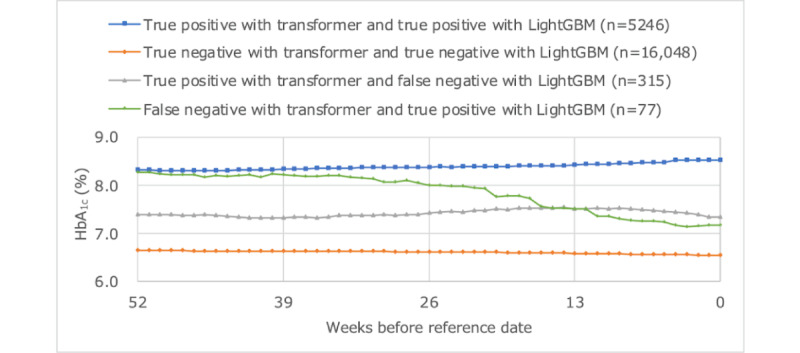
Average levels of HbA_1c_ time series. HbA_1c_: hemoglobin A_1c_.

**Figure 10:**
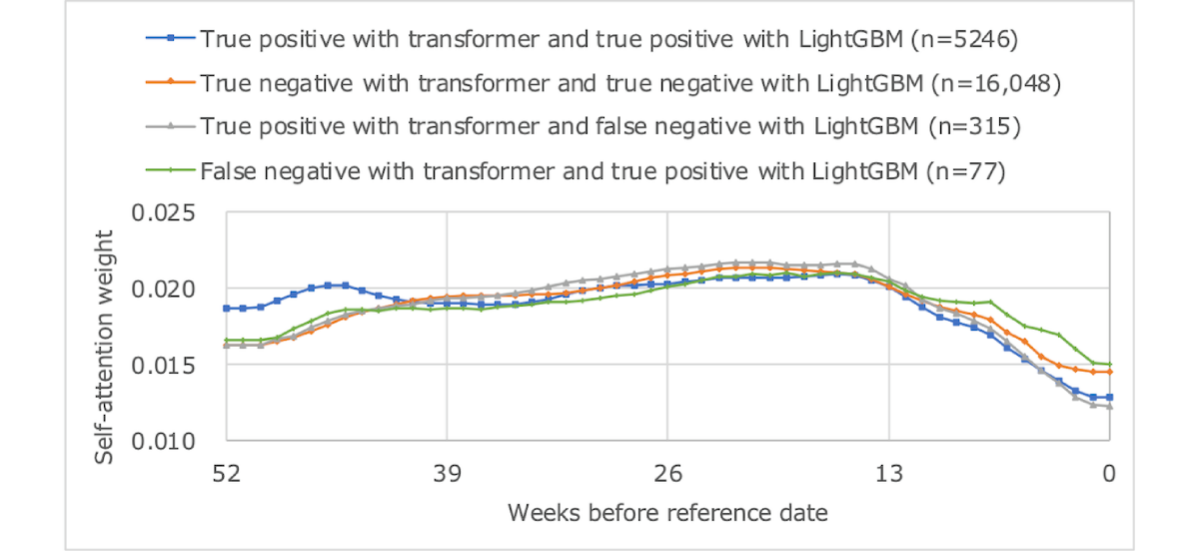
Average weight of self-attention.

**Figure 11:**
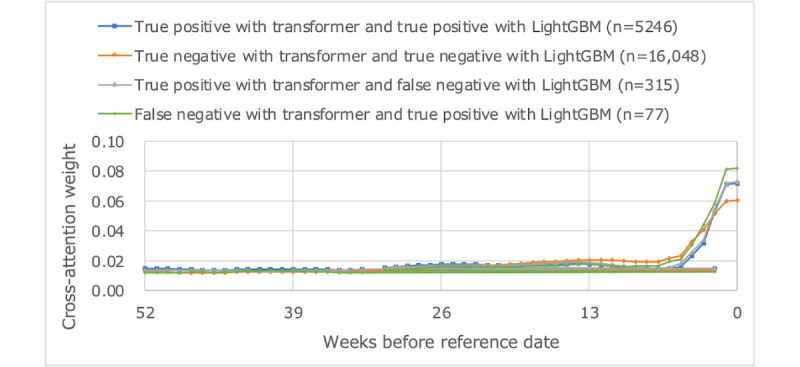
Average weight of cross-attention.

Figures 9-11 plot the average values of HbA_1c_ levels, self-attention weights, and cross-attention weights for 4 groups: true positives with transformer and true positives with LightGBM, true negatives with transformer and true negatives with LightGBM, true positives with transformer and false negatives with LightGBM, and false negatives with transformer and true positives with LightGBM. The group with true positive results in both models had an average HbA_1c_ level of 8% or higher, whereas the group with true negative results in both models had an average HbA_1c_ level of less than 7%. The weight of older self-attention was larger in the former group, and the weight of recent cross-attention was smaller in the latter group. The group containing true positives by transformer and false negatives with LightGBM had an average HbA_1c_ level of around 7.5%, had a smaller recent self-attention weight than the other groups, and had a similar trend of cross-attention weights as the group of true negatives with both models. The group that was false negative with transformer and true positive with LightGBM tended for HbA_1c_ to fall from the 8% range to the 7% range, and both recent self-attention and cross-attention were greater than other groups.

## Discussion

### Evaluation of the Predictive Accuracy

Our results show that, despite usual care by specialist physicians, poor glycemic control was common, affecting 57.83% (4504/7787) of patients. By highlighting cases with a high likelihood of poor glycemic control despite normal treatment intensifications, the proposed model provides new information to physicians, identifying patients who may benefit from extraordinary treatment intensification.

Balancing high predictive accuracy with interpretability is vital for acceptance by patients and physicians. The proposed model achieved impressive predictive accuracy, with ROC-AUC above 0.9, PR-AUC above 0.8, and an overall accuracy of 0.864. For physicians, ROC-AUC above 0.9 suggests excellent performance in distinguishing between patients who will have poor glycemic control and patients who will have good glycemic control. Similarly, PR-AUC above 0.8 indicates excellent performance in providing accurate prediction while minimizing false positives. LightGBM, a widely respected model in ML, serves as a benchmark. The proposed model slightly surpassed the performance of LightGBM, implying that the proposed model can offer physicians a reliable tool for predicting poor glycemic control.

Accuracy did not increase with longer training data lengths. The model achieved accurate predictions with just 1 year of training data, suggesting that recent glycemic control plays a dominant role in prediction outcomes. However, the actual future glycemic control is influenced by factors not accounted for in the current model, such as medications, exercise, diet, and other lifestyle factors.

While the proposed model demonstrated comparable predictive accuracy to LightGBM within this experiment’s scope, further improvement may be possible with extensive training data. Transformer models, known for power-law characteristics, benefit from scale-ups [51], and expanding this study to multiple hospitals could explore potential performance enhancements and test the applicability of the power-law in the medical field.

### Interpretability

The cross-attention weights were very similar for the group that was true positive in both models and the group that was true positive in transformer and false negative in LightGBM. This suggests that the proposed model consistently made predictions by capturing sufficient features, while the benchmark LightGBM might have captured extraneous features. On the other hand, when the proposed model performed worse than LightGBM, as observed in the group of false negatives with the transformer and true positives with LightGBM, it appears that the cross-attention strongly responded to the decreasing trend in HbA_1c_, leading to a prediction failure. These prediction failures accounted for only 0.30% (77/25,564) of cases.

### Limitations

Our study has notable limitations. First, the data were sourced from past records at a single hospital, limiting generalizability. We have not confirmed prediction accuracy for new patients, as we used a rolling origin procedure. While we separated the data into training and testing sets based on time duration, some patients still overlap between these sets. While this approach is useful for assessing the model’s performance within the hospital where it is trained, it poses challenges when applying a model trained in one hospital to another. The intensification of treatment may depend on factors specific to individual patients, the treatment strategies of individual physicians and hospitals, guidelines, and varying treatment trends across countries. Further work is needed to verify the extent to which the model needs to be customized for different environments.

Second, ML reflects majority characteristics, potentially limiting applicability to diverse patient populations. In the data set used in the experiment, as shown in Table 2, 40% of patients have 7 diseases, and patient characteristics are biased. Prediction failure analysis needs to be further scrutinized, including versus patient characteristics. We should examine this issue by comparing prediction accuracy for each patient cluster.

Third, the model uses only HbA_1c_ levels as inputs. We incorporated prescription and other laboratory tests as explanatory variables during preliminary validation, but both our proposed model and LightGBM did not show improved predictive accuracy. Future work should further explore incorporating clinical data beyond HbA_1c_. EHRs contain patient history represented in categorical, numeric, text, and images that are still underused. We should devise model designs based on cutting-edge multimodal modeling using the transformer [52-54].

Fourth, the interpretability of the model expresses temporal relationships numerically, lacking readability. To enhance clarity and visualization of the information that physicians require, it is essential to solidify the user interface or user experience concepts. There is a need for further consultation with physicians to determine an interface that would effectively communicate interpretability. Additionally, to increase the interpretability of this method, an approach that combines it with traditional XAI technologies [36] such as SHAP and LIME should be investigated.

Fifth, this was a backward-looking study, using past data, and the essential next phase is to assess the model’s predictive capabilities in clinical practice. There is a need for a careful exploration of the model’s effectiveness in real clinical scenarios.

### Future Research Direction

Our ultimate goal is to improve the treatment outcomes of diabetes. Merely predicting poor glycemic control alone cannot achieve this goal. By providing predictive results to physicians and reinforcing treatment, we can demonstrate the value of the predictions. Future research could focus on improving predictions by incorporating additional clinical data beyond HbA_1c_ levels. Exploring the applicability of the model in diverse populations will help assess its generalizability and institution-specific variations. Implementing the model in clinical practice for real-time predictions, possibly through randomized controlled trials, would elucidate its impact on clinical decision-making and patient outcomes. Moreover, expanding the scope to predict the impact of treatment changes as well [55] could further enhance the model’s utility in diabetes management.

### Conclusions

The proposed model addresses the challenge of identifying patients with T2D who will have poor glycemic control, increasing the risk of complications, despite usual care by specialist physicians. The model achieves highly accurate predictions, with an accuracy of 0.864, and provides good interpretability from the irregularly spaced HbA_1c_ values commonly observed in clinical settings. The model balances desirable predictive accuracy and interpretability in clinical practice, enhancing the acceptability of ML. Future efforts should focus on further improving accuracy and interpretability by incorporating additional features beyond HbA_1c_ and validating large clinical data sets.

## Abbreviations

AUC: area under the curve
EHR: electronic health record
HbA_1c_: hemoglobin A_1c_
ML: machine learning
PR: precision-recall
ROC: receiver operating characteristic
T2D: type 2 diabetes
XAI: explainable artificial intelligence

## Data Availability

The data in this study are not openly available because of the restrictions imposed by the research ethics committees that approved this study.
